# Does the effectiveness of core stability exercises correlate with the severity of spinal stenosis in patients with lumbar spinal stenosis?

**DOI:** 10.12669/pjms.333.12123

**Published:** 2017

**Authors:** Chaxiang Chen, Zhichao Lin, Yingjie Zhang, Zemin Chen, Shujie Tang

**Affiliations:** 1 Chaxiang Chen, Medical Image Center, The First Affiliated Hospital, Jinan University, Guangzhou, 510632, China; 2 Zhichao Lin, Medical Image Center, The First Affiliated Hospital, Jinan University, Guangzhou, 510632, China; 3 Yingjie Zhang, Department of Pain, Qingzhou Hospital of Traditional Chinese Medicine, Qingzhou, Shandong Province, 262500, China; 4 Zemin Chen, Medical Image Center, The First Affiliated Hospital, Jinan University, Guangzhou, 510632, China; 5 Shujie Tang, College of Traditional Chinese Medicine, Jinan University, Guangzhou, 510632, China

**Keywords:** Lumbar spinal stenosis (LSS), Core stability exercises (CSE), Japanese Orthopaedic Association score (JOA), Self-reported walking distance

## Abstract

**Objective:**

To determine whether the effectiveness of core stability exercises correlates with the severity of spinal stenosis in patients with degenerative lumbar spinal stenosis.

**Methods:**

Forty-two patients with degenerative lumbar spinal stenosis treated in the department of orthopedics of our hospital between May 2013 and January 2016 were included in the study. All the patients performed core stability exercises once daily for six weeks, and the clinical outcomes were evaluated using Japanese Orthopaedic Association (JOA) score and self-reported walking capacity. The anteroposterior osseous spinal canal diameter was measured to evaluate the severity of spinal stenosis. The correlation between the stenosis degree and the differences of Japanese Orthopaedic Association score or self-reported walking capacity at baseline and after treatment were analyzed.

**Results:**

The patients were divided into three groups according to the spinal stenosis degree. In the three groups, there was no significant difference in JOA or self-reported walking distance at baseline (p>0.05) and after treatment (p>0.05). The JOA scores and self-reported walking distance were significantly increased after treatment (p<0.05) in any of the three groups when compared to the baseline. Also, there was no significant correlation between the stenosis degree and the difference of JOA (p>0.05) or self-reported walking distance (p>0.05).

**Conclusion:**

There was no significantcorrelation between the effectiveness of core stability exercises and the severity of spinal stenosis in patients with degenerative lumbar spinal stenosis.

## INTRODUCTION

Lumbar spinal stenosis (LSS) is a common spinal disorder in old people,^1^ its incidence is as high as 30%.^2^ With the aging society, more and more old adults may suffer from the disease, which affects the life quality of patients and exerts a heavy burden on social security systems. Conservative treatment is the primary option for LSS,^3^ there is no significant difference in long-terms efficacy between conservative and surgical treatment.^4^ Moreover, some authors suggest that neither the clinical manifestations nor the efficacy of conservative treatment is significantly correlated with the severity of spinal stenosis.^5^-^7^

Among the conservative treatment, the efficacy of muscle exercises has been confirmed by many authors.^8^,^9^ In the recent decade, core stability exercises (CSE) have been performed widely in rehabilitation of low back pain. It has a positive effect on pain relief and trunk stability improvement, facilitating skilled motor behavior and daily activities.^10^ In a study of 102 patients with LSS, Zhang found CSE could relieve the pain and improve the quality of life of patients.^11^ We speculate that the efficacy of CSE in the treatment of LSS may also have no significant correlation with the severity of spinal stenosis. While, few studies have been published on the issue in English literatures.

Therefore, we reviewed the forty-two patients with LSS treated using CSE in orthopedics department of our hospital between May 2013 and January 2016, the objective of our study was to evaluate the efficacy of CSE in treating LSS, and determine the correlation between the efficacy and severity of spinal stenosis in patients with LSS.

## METHODS

The patients treated using CSE in the department of orthopedics of our hospital between May 2013 and January 2016 were included in the study. The inclusion criteria included: (1) Neurogenic intermittent claudication; (2) narrowed lumbar spinal canal, nerve root canal or intervertebral foramen confirmed by MRI; (3) the patients with ability to communicate and cooperate with medical workers;^12^ (4) to facilitate the study, only patients with L4 stenosis were included. Those patients with cauda equine syndrome, Paget’s disease, severe osteoporosis or metastasis to the vertebrae, significant scoliosis (Cobb angle>25°), previous laminectomy, degenerative or lytic spondylolisthesis or significant instability of lumbar spine, and severe comorbidity that increased the risk to the patients or interfered with the assessment of the study were excluded.^12^ This study was approved by the Ethics Committee of our hospital, and all the participants provided written informed consent.

All the participants performed CSE including plank, side plank, bridge, straight leg raise and modified push-up, each movement was carried out ten times for one arm/leg, once daily for six weeks.^10^ The clinical outcome was evaluated using Japanese Orthopaedic Association (JOA) score and self-reported walking distance at baseline and after treatment. The JOA score is composed of subjective symptoms, clinical signs, impairment of activities of daily living, and urinary bladder function.^13^ Walking is an important daily functional measure^14^ and intermittent claudication is the hallmark symptom for LSS, so self-reported walking distance was used to evaluate the outcomes.

To evaluate the severity of spinal stenosis, the smallest of the anteroposterior spinal canal diameters was used,^7^ which was measured on T1-weighted MR images using Image J. To classify the degree of spinal stenosis, the diameter less than 15 mm and more than 12 mmm was defined as I° stenosis, less than 12 mm and more than 10 mm was defined as II° stenosis, and diameter less than 10 mm defined as III° stenosis.^5^

Statistical analysis was carried out using SPSS21.0 (SPSS Inc., Chicago, IL, USA). The intragroup comparisons of JOA or self-reported walking distance were carried out using paired t test, and the intergroup comparisons using Analysis of Variance. The correlation between variables were evaluated using Pearson correlation analysis. A P value less than 0.05 indicates statistical significance.

## RESULTS

Forty-two patients were included in the study. According to the outcomes of MR measurement, I°, II° and III° stenosis were detected in 15, 17 and 10 patients, and the patients were assigned into I°, II° and III° stenosis group, respectively. Before and after treatment, there was no significant difference in JOA scores and self-reported walking distance among the three groups (p>0.05). After treatment, both the JOA scores and self-reported walking distance were significantly increased (p<0.05) in any of the three groups when compared to the baseline (Table-I).

**Table-I:** The comparison of JOA and SRWD in three groups.

	*JOA*		*SRWD(m)*	

	*At baseline*	*After treatment*	*At baseline*	*After treatment*
I° stenosis	14.24±3.04	21.35±3.18^a^	415±193	734±259^a^
II° stenosis	13.67±2.80	21.33±3.23^a^	426±243	711±322^a^
III° stenosis	12.44±2.59	20.89±2.33^a^	448±202	790±247^a^

SRWD=self-reported walking distance.

a denotes p<0.05 in comparison to baseline.

**Fig.1:**
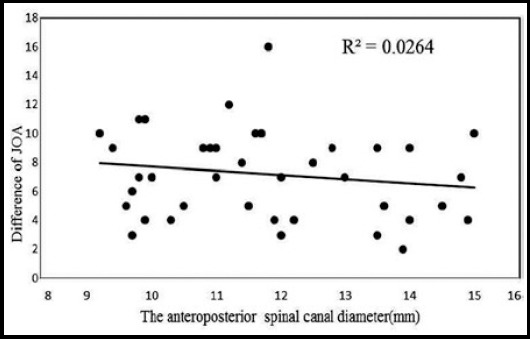
The distribution of the difference of JOA in patients.

**Fig.2:**
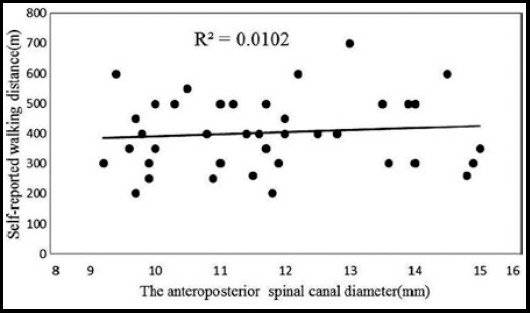
The distribution of the difference of self-reported walking distance in patients.

The difference of JOA scores after treatment and at the baseline is listed in Fig.1, Pearson correlation analysis showed the correlation coefficient was -0.162 and p value was 0.304, there was no significant correlation between the difference of JOA and spinal stenosis degree (p>0.05) (Fig.1). The difference of self-reported walking distance after treatment and at the baseline is listed in Fig.2. Similar as JOA, Pearson correlation analysis showed the correlation coefficient was 0.101 and p value was 0.524, no significant correlation was found between the difference of self-reported walking distance and spinal stenosis degree (p>0.05

## DISCUSSION

In the current study, we tried to evaluate the efficacy of CSE in the treatment of LSS as well as the correlation between the efficacy and severity of spinal stenosis. This study may help physicians better understand the treatment of LSS. To the best of our knowledge, few studies have been published in this regard in English literatures.

We found, after treatment, the JOA and self-reported walking distance increased in the included patients in comparison to the baseline. This indicates CSE can play an active role in relieving pain, and improving daily activities for patients with degenerative LSS. The results confirmed the conclusion of many scholars.^3^,^8^,^9^ In terms of the mechanism of CSE in treating LSS, we attribute it to its effect on lumbar alignment. Yagi analyzed 120 patients with degenerative LSS, and found there was causal relationship between paravertebral muscle and global spine alignment.^15^ Abbas studied 167 individuals with or without the symptoms of degenerative LSS, found lumbar lordosis and sacral slope were significantly smaller in the individuals with symptoms than those without.^16^ Moreover, some studies have confirmed the effect of CSE on paravertebral muscles.^17^ In the current study, after treatment, the JOA and self-reported walking distance significantly increased, demonstrating that CSE may increase the activation of deep fibers and cross-sectional area of paravertebral muscles, improve the stability and coordination of lumbar spine,^10^ adjust the lumbar alignment and subsequently the symptoms were improved.

In addition, we found between the three groups there was no significant difference in JOA scores or self-reported walking distance at baseline or after treatment. The symptoms of LSS often poorly correlate with their radiological findings and many asymptomatic persons even showed severe narrowing of spinal canal in MRI.^18^ In a multi-center cohort study, Burgstaller didn’t find any correlation between MRI findings and the severity of symptoms either.^6^ In the current study, we had the similar results.

At the same time, we found there was no significant correlation between the stenosis degree and the difference of JOA scores or self-reported walking distance, this indicates that the spinal stenosis degree doesn’t correlate with the efficacy of CSE in treating degenerative LSS. Some authors also have the same conclusion in their studies on conservative treatment of degenerative LSS, but they performed other conservative methods instead of CSE^5^. Although some authors suggest that there is a need for innovative methods or techniques to detect the causal relationship between radiological findings and the complaints of patients with LSS,^6^ we attributed the clinical outcomes to the same effect of CSE on lumbar alignment in this study.

### Limitations of the study

First, we suggest that CSE may adjust the lumbar spine alignment by improving the muscle forces, but we didn’t carry out a comparative measurement of lumbar lordosis or sacral slope. Second, JOA and self-reported walking distance are subjective measures, in which the subjectivity of the patients may influence the final results adversely. Subsequently, more studies need to be carried out in the future.

Despite of the limitations, we conclude that CSE can relieve the pain and improve the daily activities of patients with degenerative LSS, but its efficacy is not significantly correlated with the severity of spinal stenosis.
